# Macrophages in chronic infections: regulation and remodeling

**DOI:** 10.3389/fimmu.2025.1594988

**Published:** 2025-07-17

**Authors:** Hongle Cui, Min Wang, Sitan Jiao, Sirui Tian, Hui Liu, Bo Luo

**Affiliations:** ^1^ Department of Parasitology, Zunyi Medical University, Guizhou, China; ^2^ School of Basic Medicine, Zunyi Medical University, Guizhou, China; ^3^ School of Stomatology, Zunyi Medical University, Guizhou, China

**Keywords:** macrophages, polarization, metabolic reprogramming, chronic infection, immune regulation

## Abstract

Macrophages, as a critical component of innate immune cells, exhibit significant plasticity. When confronted with danger signals such as pathogens or microenvironmental alterations, macrophages can differentiate into various phenotypes and functions to safeguard the host. However, numerous pathogens manipulate macrophage metabolic pathways to modify their functional expression, facilitating immune evasion and ensuring long-term survival during chronic infections. Therefore, the role of macrophage metabolic reprogramming in chronic infections has received growing attention. This review elucidates the primary metabolic pathways of macrophages and their association with polarization. It examines how pathogens modulate macrophage functional expression through metabolic reprogramming to sustain chronic infection. Additionally, it delineates how macrophage metabolic reprogramming in chronic infections reconfigures the microenvironment through interaction with other immune cells and its contribution to trained immunity.

## 1 Introduction

Macrophages exhibit significant plasticity and can differentiate into various phenotypes and functions when confronted with pathogen infections or alterations in the microenvironment (1, 2). This versatility and adaptability of macrophage functions become especially prominent during chronic infections. Traditionally, the proliferation of pathogens and the expression of cellular functions in chronic infections have mainly relied on metabolism to meet energy requirements. However, metabolites are increasingly recognized to have key functions in intracellular, intercellular, and interorgan communication to regulate signaling and organ function (3). In recent years, the bidirectional dynamic process of macrophage metabolic reprogramming and polarization has been elucidated, gradually uncovering how macrophages balance host defense and pathogen immune evasion, and in this way, it provides new treatment for chronic infectious diseases (3–5). Therefore, in this review, we will illustrate the effect of alterations in the metabolic microenvironment on macrophage polarization in chronic infection from the perspective of infected organs.

As a pivotal cell type in innate immunity, macrophages rapidly respond to pathogens in the early stages of infection through their intrinsic phagocytic abilities and antigen-presenting functions. The functions of macrophages are determined by the polarization state (M1 or M2) (6). In response to pathogen invasion,M1 macrophages are characterized by high expression of cluster of differentiation (CD)80, and CD86, and secrete various pro-inflammatory cytokines, such as interleukin (IL)-1β, IL-6, IL-12, and nitric oxide synthase 2 (iNOS) to exacerbate the inflammatory response (7). In contrast, M2 macrophages are characterized by high expression of the mannose receptor (CD206) and scavenger receptor (CD163), and secretion of arginase-1, IL-10 to limit inflammation and tissue repair (8).

However, their roles extend far beyond these functions; macrophages can polarize into M1 or M2 types under the influence of microenvironmental factors, exhibiting either pro-inflammatory or anti-inflammatory states. In the complex microenvironment of the body, typical macrophage polarization phenotypes encompass not only the M1 phenotype induced by lipopolysaccharides (LPS) and the M2 phenotype induced by IL-4 or IL-13, but also further subdivisions of the M2 subgroups based on their specific function (9). These subgroups include the M2b type, activated by immune complexes and TLR ligands to produce pro-inflammatory factors; the M2c type, activated by glucocorticoids or IL-10 to primarily exert anti-inflammatory functions; and the M2d type, also known as TAMs (Tumor-Associated Macrophages), activated by TLR ligands and A2 adenosine receptor agonists, which play key roles in regulating tumor progression, angiogenesis, and metastasis (10).

Studies have identified substantial alterations in intracellular metabolic pathways during M1 and M2 polarization of macrophages, supplying the essential energy and molecular framework for their functional transitions (11). During the early stages of chronic infection, macrophages polarize into the pro-inflammatory M1 type to combat pathogen invasion. However, as the infection progresses, pathogens secrete derived factors or metabolic products that modify the metabolic pathways of host macrophages, resulting in functional alterations (11). Consequently, in the later stages of infection, macrophages shift from the M1 type to the anti-inflammatory M2 type, facilitating pathogen immune evasion.

Macrophages primarily generate energy through five metabolic pathways: glycolysis, the tricarboxylic acid (TCA) cycle, the pentose phosphate pathway (PPP), fatty acid metabolism (including fatty acid oxidation [FAO] and fatty acid synthesis [FAS]), and amino acid metabolism. The main steps of glycolysis occur in the cytoplasm, where glucose is broken down into pyruvate under aerobic conditions (12). Some pyruvate is converted to lactate and exported out of the cell, while some is converted to acetyl-CoA to participate in the TCA cycle. Under anaerobic conditions, glucose is broken down into lactate and ATP is produced. The TCA cycle, also known as the Krebs cycle or citric acid cycle, occurs in the mitochondria. Acetyl-CoA involved in the cycle can originate from three sources: pyruvate from glycolysis, fatty acyl-CoA from fatty acid metabolism, and acetate from either acetate metabolism or extracellular uptake. The primary products of the TCA cycle are NADH and FADH2, which are transferred to the electron transport chain to support oxidative phosphorylation (OXPHOS) and the efficient generation of ATP (13). Lipid metabolism pathways primarily involve fatty acid oxidation, fatty acid synthesis, and fatty acid uptake. Amino acid metabolism is also indispensable in macrophages; for example, glutamine enters cells through various Slc transport proteins and is converted to glutamate. Glutamate can then generate glutathione, which helps control redox balance, or be converted to α-ketoglutarate to enter the TCA cycle (14). These five metabolic pathways exhibit metabolic adaptability in macrophages depending on the varying microenvironments they encounter, thereby influencing the different functional phenotypes of macrophages.

When facing external pathogens or signals, such as during tissue repair, macrophages undergo metabolic reprogramming to produce distinct polarized phenotypes, granting them high plasticity. Unlike resting macrophages, which rely on oxidative phosphorylation for energy, M1 polarized macrophages exhibit increased glycolytic metabolism, an interrupted tricarboxylic acid (TCA) cycle, activation of the pentose phosphate pathway (PPP), and fatty acid synthesis (FAS) as their primary metabolic modes. The enhanced glycolytic metabolism in M1 macrophages sustains the energy demands for their pro-inflammatory functions and, through the PPP, provides intermediates necessary for amino acid and nucleotide synthesis (15). The PPP is also the main source of nicotinamide adenine dinucleotide phosphate (NADPH), which is used to produce reactive oxygen species (ROS) and nitric oxide (NO) (16). Additionally, the disruption of the TCA cycle leads to the accumulation of TCA intermediates, such as citrate, itaconate, and succinate, which sustain the inflammatory response by increasing the production of lipid mediators, prostaglandins, NO, and ROS. High levels of succinate can stabilize hypoxia-inducible factor 1-alpha (HIF1-α) and induce the secretion of pro-inflammatory cytokines, such as IL-1β.

The polarization changes of macrophages during the host immune response to chronic infection reflect their complexity in maintaining tissue homeostasis and regulating immune responses. During the dynamic polarization process induced by chronic infection, macrophages experience a period of oxidative stress following an initial burst of oxidation and then undergo metabolic rebalancing, transitioning from M1 to M2 polarized macrophages. This phase is characterized by the reconfiguration of the TCA cycle and the regulation of enzyme activities related to metabolic pathways. In addition, macrophage metabolism shifts towards oxidative phosphorylation (OXPHOS), fatty acid oxidation, and glutamine metabolism (17). Macrophages promote fatty acid oxidation, generating acetyl-CoA from long-chain fatty acids, which is then oxidized in the TCA cycle and electron transport chain (ETC) to produce energy, or transferred to the cytoplasm to regulate NADPH. Glutamine metabolism plays a crucial role in sustaining the TCA cycle by providing carbon and nitrogen for the synthesis of amino acids, proteins, nucleotides, and lipids (18). Furthermore, the literature indicates that α-ketoglutarate derived from glutamine catabolism has the potential to limit M1 polarization by inhibiting the NF-κB pathway (19).

Macrophages are involved in metabolizing in a way that is specific to other APCs. It has been reported that EBNA2-driven NAD *denovo* synthesis via kynurenine metabolism critically regulated respiration during this early phase of infection (20). But it is no doubt that the balance between aerobic glycolysis and mitochondrial respiration is an important regulator of B cell activity in different states of differentiation (21). In addition, dendritic cells (DCs), as antigen-presenting cells, rely on oxidative phosphorylation supported by lipid breakdown in the microenvironment for energy at rest (22). In the face of chronic infection, the DC undergoes two metabolic reprogramming for activation; Early glycolysis is rapidly upregulated, which in turn supports other metabolic processes such as fatty acid synthesis (FAS) and lipid synthesis, which in turn promote lymphocyte activation. In the later stages of dendritic cell activation, iNOS-dependent glycolysis is performed (23).

## 2 The impact of metabolic reprogramming on macrophage polarization phenotypes in chronic infections

Chronic infections are persistent pathogen infection states that typically last for months or years, resulting in sustained inflammatory responses. Macrophages play a critical role in chronic infections as key components of the immune system. They are responsible for phagocytosing and digesting pathogens, clearing cellular debris at infection sites, and releasing cytokines and chemokines to regulate the immune response. Although macrophages can control many microorganisms, they do not always exhibit microbicidal activity. Various pathogens exploit macrophages as intracellular microenvironments for development, replication, or evasion from other immune cells.

**Figure 1:**
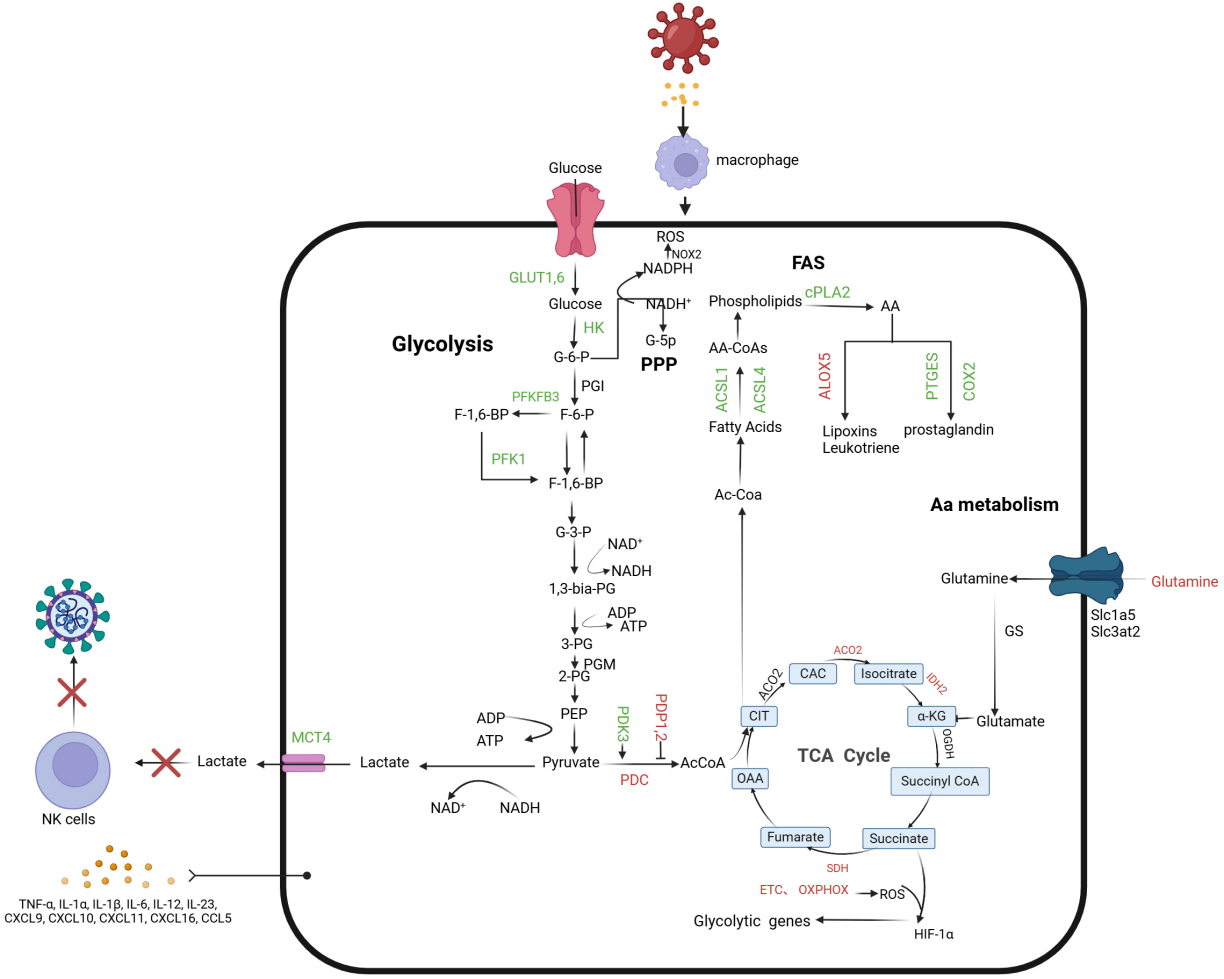
Macrophage-associated metabolic reprogramming in early chronic infection. During macrophage infection, glycolytic flux increases, leading to lactate formation and secretion, while mitochondrial oxidative metabolism decreases. This includes reduced pyruvate oxidation by the Pyruvate Dehydrogenase Complex (PDC) and downregulation of the TCA cycle and oxidative phosphorylation. Key regulators of glycolysis, such as PFKFB3, glucose transporters (GLUT1 and GLUT6), and glycolytic enzymes (HK1, HK2, PFK-1), are upregulated. The Warburg effect is evident through elevated Lactate Transporter Member 4 (MCT4) and increased HIF-1α activity, driven by ROS from the ETC and succinate accumulation in the TCA cycle. Citrate, a TCA intermediate, supports fatty acid and prostaglandin synthesis. Increased ACSL1 and ACSL4 levels enhance long-chain fatty acid incorporation into membrane phospholipids, while elevated cPLA2 releases arachidonic acid (AA), promoting prostaglandin production via COX2 and PTGES. Excessive lactate inhibits NK cell glucose utilization and creates an acidic microenvironment, impairing NK cell function. Green arrows: promotion; red arrows: inhibition. PDK 3, Pyruvate dehydrogenase kinase 3; PDP 1-2, pyruvate dehydrogenase phosphatases 1 and 2; PDC, Pyruvate dehydrogenase complex; ACO2, aconitase 2; ACOD 1, Aconitate decarboxylase 1; IDH 2, Isocitrate dehydrogenase 2; SDH, Succinate dehydrogenase; G-6-P, glucose 6-phosphate; F-6-P, fructose 6-phosphate; G-3-P, Glyceraldehyde 3-phosphate; 1, 3-Dual-PG, 1, 3-di-phosphoglycerate; 3-PG, 3-phosphoglyceride; 2-PG, 2-phosphoglyceride; PEP, Phosphoenolpyruvate; AcCoA, acetyl-CoA; OAA, Oxaloacetic acid; CIT, Citrate; CAC, Metaconic acid; α -KG, α -Keto-ketoglutarate; FUM, Fumarate.

The polarization and metabolic reprogramming of macrophages under chronic infection conditions are essentially dynamic adjustment processes that balance energy supply and demand. Changes in energy metabolism not only provide macrophages with sufficient ATP to sustain their activities and survival, but are also closely related to cell polarization, regulating immune function (**Figure 1**). In the microenvironment of some pathogen-induced chronic infections, pathogens can often manipulate the host macrophages’ metabolic pathways, affecting the expression of their polarization phenotypes and inhibiting their pro-inflammatory responses, thereby achieving immune evasion. In this context, further elucidation of the relationship between macrophage metabolic reprogramming and polarization in chronic infections is crucial for understanding the complexity of host-pathogen interactions.

Immunometabolism refers to how the metabolism of immune cells is influenced by the complex internal microenvironment during their functioning and differentiation, thereby altering their fate. In the host immune response to chronic infections, macrophage polarization changes reflect their complexity in maintaining tissue homeostasis and regulating immune responses. Various cytokines and chemokines in the microenvironment can effectively promote the transition of macrophages from the M1 to the M2 phenotype (24). As the disease progresses, the pathogen-killing and pro-inflammatory functions of M1 macrophages, predominant in the early stages of infection, gradually weaken. In contrast, the roles of M2 macrophages in tissue repair, fibrosis formation, and immune response regulation are enhanced. This transition is a dynamic balancing process closely related to the type of infection, pathogen characteristics, and host status, demonstrating that the polarization state of cells precisely reflects the dynamic immune microenvironment during chronic infection.

In chronic infections such as tuberculosis and HIV, polarized macrophages secrete various immunoregulatory factors, such as IL-10 and TGF-β, which not only participate in the maturation and fibrosis process of lesions but may also establish an immunosuppressive microenvironment conducive to pathogen survival (25). Metabolic reprogramming of macrophages during their dynamic polarization process is crucial, as pathogens manipulate this reprogramming to influence macrophage polarization (26). In the late stages of tuberculosis, the metabolic status of infected macrophages, characterized by glucose uptake inhibition, suppression of glycolysis, and the restoration of TCA cycle activity and oxidative phosphorylation, indicates macrophage adaptation to pathogen invasion. In HIV infection, macrophages undergo specific metabolic adaptations, such as increased glucose uptake and glycolysis, which aid in viral replication and dissemination. These metabolic shifts, which are key features of macrophage polarization, offer new perspectives for understanding the immunoregulation of chronic infections and potential therapeutic targets for disease treatment.

Analyses of macrophages in differing organ environments highlight their metabolic plasticity and its link to their functions. Tissue-resident macrophages (TRMs) are specific and require specialized cellular metabolism to maintain homeostasis. Peritoneal, spleen, and hepatic TRMs showed higher GLUT 1, PKM, and G6PD expression compared to other TRMs, indicating active glucose uptake, glycolysis, and the pentose phosphate pathway (PPP) (27). Lung, spleen, liver, and peritoneal TRMs express CD36 that mediates lipid uptake, while peritoneal TRMs show the highest CPT 1A and ACC 1 expression, Microglia express only significant amounts of GLUT 1 (27). Indeed, peritoneal TRMs take up and metabolize glucose more efficiently than do lung AMs (27). And due to the different ways in which cells metabolize, the functions they express are not consistent (28). Therefore, this article will focus on the expression of metabolic modes and functions of macrophages residing in different tissues in the context of chronic infection.

### 2.1 Alveoli macrophage

In the resting state of alveoli macrophage (AM), macrophages resident in the lungs have the ability to process from cholesterol to the breakdown of fatty acids, phospholipids, and other lipids and anabolic lipids, as well as to maintain their lipid and cholesterol processing activity for high basal respiration, but they are less involved in glycolysis (29–32). In addition, AM recycles lipid-rich lung surfactants and regulates inflammatory responses, so AM is more dependent on lipid metabolism as a pathway for its own energy function (32). Interstitial macrophages (IMs) are also present in the lungs, but they are not TRMs and rely primarily on glycolytic function. In the face of chronic infection, these two subsets of macrophages exhibit different inflammatory states due to different metabolic programs after infection. AM primarily maintains anti-inflammatory expression of fatty acid oxidation (FAO) and oxidative phosphorylation (OXPHOS), while IMs exert a pro-inflammatory response through glycolysis to maintain host defense (33). Recent studies have shown that several changes in fatty acid metabolism in *Mycobacterium tuberculosis*(Mtb)-infected AM have recently been identified (34). AM utilizes FA induced by PPAR-α and has a lower burden of Mtb infection compared to mesenchymal macrophages during glycolysis-dependent Mtb infection (33, 34), showed in **Table 1**. In addition, SARS-CoV-2 infection leads to the activation of inflammasomes and the excessive release of IL-1β by affecting peroxidases in AM (which have higher expression of related genes compared to other lung macrophages), organelles that play an important role in lipid metabolism and redox balance (35). However, although targeted drugs are made for lipid metabolism in AMs, the therapeutic effect is not ideal (36). So glycolysis and iron metabolism of AMs should be the direction of our future research.

**Table 1:** Specific metabolic changes and functional expression of different tissues in chronic infection.

Chronic infection	Macrophage subsets	Metabolic change	Functional change
Mycobacterium tuberculosis (Mtb)	Alveolar macrophages (AM)	FAO↑, Lipid accumulation↑	Host response↑, Bacterial burden↓
	Interstitial macrophages (IM)	HIF1a Warburg shift↑, glycolysis genes↑	IL-1β and bacterial killing↑
SARS-CoV-2	Alveolar macrophages (AM)	Peroxidases↓	IL-1β and inflammasomes↑
Schistosoma japonicum	Liver Kupffer cells (KC)	Glycolysis and FAO↑, Fas and Cd36↓	IL-10↑
Influenza infection	Liver Kupffer cells (KC)	Fas↑	ROS ANDIL-6↑
Ulcerative colitis	Lamina propria-associated macrophages (LAMs)	FAO and OXPHOS↓ glycolysis↑	Pro-inflammatory factors↑
EBV	Intestinal macrophages (IMs)	Glycolysis↑	NLRP3↑
HPV	Liver Kupffer cells (KC)	Glycolysis and FAO↑, Fas and Cd36 ↓	IL-10↑

↑ means increase and ↓ represents down.

### 2.2 Liver Kupffer cell

Liver Kupffer cells (KC) rely on transcription factors to regulate the active metabolism of iron (Spi-C and NRF2) as well as lipids (PPARγ, LXRα, and SREBP1) to maintain homeostasis in the spleen and liver (37). A study in mouse model of *Schistosoma japonicum* infection showed that macrophages infiltrating liver tissue were activated by soluble egg antigen (SEA), leading to the upregulation of genes related to glycolysis and fatty acid oxidation, thereby promoting M2 functional polarization (38), showed in **Table 1**. Furthermore, In influenza infection, KC can kill hepatocytes directly through the Fas-dependent apoptotic pathway, or indirectly by stimulating cytokine secretion and other mediators (e.g., ROS) to interact with CD8+ (and possibly CD4+) T cells (39). Interestingly, Kupffer cells and splenic macrophages show functional similarities, in comparison with peritoneal macrophages, as reflected by comparable levels of TLR4, TLR7/8, and TLR9 mRNA and low or undetectable levels of TNF and IL-12p40 produced upon TLR ligation. What role metabolism plays in this is worth exploring in the future (40).

### 2.3 Intestinal macrophage

Intestinal macrophages (IMs) have several subpopulations (41). They can ameliorate colitis and support the proliferation of colonic epithelial cells by providing polyamines, which IMs synthesize in an mTORC1-dependent manner (42). In general, lamina propria-associated macrophages (LAMs) have lower levels of expression of FAO-related genes compared to epithelial cell-associated macrophages in the human colon (43). LAMs in patients with inflammatory bowel disease further downregulated genes that control fatty acid metabolism and mitochondrial OXPHOS, while upregulating glycolytic genes, compared to LAMs from healthy donors (43). Based on research, EBV can enhance the glycolysis of intestinal macrophages, thereby excessively secreting inflammasomes and promoting ulcerative colitis, showed in **Table 1**.

Interestingly, the metabolic states of IMs are controlled by the composition of ingested diet and the gut microbiota (44). For example, the dietary sugar mannose reduces the proinflammatory activity of colonic macrophages during colitis in mice (45); Oral butyrate supplementation increased FAO and OXPHOS in mouse IMs, and ex vivo butyrate exposure decreased LPS-mediated pro-inflammatory activation of IMs (46); ATP from microorganisms acts on bone marrow cells to induce CSF2 production in type 3 innate lymphocytes located in isolated solitary lymphoid tissue (SILT) in the mouse gut, and this macrophage population exhibits higher mitochondrial membrane potential, enhanced ROS production, and increased expression of OXPHOS-related genes compared to LAM (47). Therefore, the study of the mechanism of homeostasis of intestinal microbiota and dietary food types on the regulation of intestinal macrophages will provide a new therapeutic direction for chronic intestinal infection-related diseases.

It is noteworthy that recent studies have identified a potential protective effect of chronic infections against obesity and sepsis. The accumulation of lactate from the pro-inflammatory response of macrophages in the early stages of infection has been shown to be detrimental to the host. Furthermore, parasites have been observed to preferentially infect dermal cells, which are involved in tissue repair. Therefore, the metabolic reprogramming of macrophages and the regulation of their polarization during chronic infections may not be solely influenced by parasites but may also involve host factors (15). While the relationship between pathogens and macrophages in chronic infections is becoming increasingly understood, further mechanistic studies are required to elucidate the interactions between pathogens and the host in macrophage metabolic reprogramming. This could potentially lead to the development of new therapeutic approaches in the future.

### 2.4 Signal pathways regulating macrophage metabolism in chronic infection environments

The molecular mechanisms of macrophage polarization in chronic infections are not yet fully elucidated. It is known to be a complex process involving the interplay of multiple factors regulated by various intracellular and extracellular signaling molecules and pathways. During the symbiosis between pathogens and the host, pathogens modulate macrophage metabolic pathways through different signaling routes, thereby affecting macrophage phenotype expression. Studies have shown that signaling pathways such as JAK2/STAT, JNK/p38, TLR4/NF-κB, and DLL4/Notch are involved in M1 macrophage polarization, while IL-4-STAT6, ERK/STAT3, and mTOR signaling pathways participate in M2 macrophage polarization (48).

M1 macrophages primarily rely on glycolysis to meet their biosynthetic and energetic demands. During chronic infections, M1 macrophages are activated via the PI3K/AKT pathway, which upregulates the expression of NF-κB, thereby inducing M1 macrophage polarization. It has been demonstrated that the downregulation of AKT1 expression results in the negative transcriptional regulation of miR-155, activation of RelA/NF-κB, inhibition of the suppressor of cytokine signaling 1 (SOCS1), and ultimately promotes M1 macrophage polarization. Simultaneously, the activated PI3K/AKT pathway upregulates multiple key glycolytic enzymes and enhances the capacity of macrophages to uptake and utilize glucose (25). G protein-coupled receptors (GPCRs), receptor tyrosine kinases (RTKs), and Toll-like/IL-1 receptors (TLR/IL-1R) all activate the PI3K/AKT pathway, augmenting cancer-associated inflammation in tumor-associated macrophages (TAMs) and advancing the glycolytic progression of M1 macrophages. Additionally, research indicates that *T.cp*-MIF secreted by the conjunctival nematode may initially activate the NF-κB pathway by binding to TLR4, promoting M1 macrophage polarization (25, 49). After 48 hours, the NF-κB pathway is inhibited, and the PI3K/Akt signaling pathway is activated, recruiting several M2 macrophage-related functional gene transcriptions, ultimately promoting M2 macrophage polarization and suppressing the host immune response. Therefore, we can see that TLRs and related pathways play crucial roles in macrophage polarization. Wang et al. (50) also found that during Staphylococcus aureus infection, TLR2 mediates the PI3K/Akt and c-Raf/MEK/ERK pathways, increasing the phosphorylation of forkhead box O1 (FoxO1) and promoting M2 polarization (25).

Peroxisome proliferator-activated receptors (PPARs) serve as pivotal sensors in lipid metabolism. Functioning as both nuclear receptors and transcription factors, PPARs are capable of directly initiating or suppressing the expression of numerous target genes, thereby regulating cellular carbohydrate and lipid metabolism. The secretion of IL-13 and IL-4 by adipocytes or Th2 cells activates STAT6 and phosphorylates AMPK, leading to an enhanced expression of PPAR-δ and ACE (15). This activation mitigates M1 polarization while promoting the expression of M2-type genes (51). Depletion of PPARγ results in the suppression of M2 macrophage polarization (52). Research indicates that in infections, M2 macrophage polarization is inhibited by arachidonic acid, whereas its metabolic derivative, prostaglandin E2 (PGE2), paradoxically promotes this process. PPARγ facilitates the linkage between these dynamics through OXPHOS (51). PGE2 enhances OXPHOS by inhibiting PPARγ, leading to selective activation of macrophages. PPARδ, a member of the PPAR family, plays a crucial role in the clearance of apoptotic cells and is involved in tumor architecture. Studies have shown that PPARα/β promotes tumor-associated macrophage (TAM) activation by augmenting the expression of IL-10 and induces the polarization process within M2 macrophages (15).Moreover, in hepatitis C virus (HCV) infection, its single-stranded RNA induces macrophage transition from M0 to M2 via TLR7, facilitating the long-term coexistence of the pathogen within the host, promoting tissue repair and remodeling, and leading to chronic HCV infection (53). Additionally, in MTB infection, cells increase PPARγ expression through a TLR2-dependent pathway. Once activated, PPARγ interacts with TR4, increasing CD36 expression, leading to lipid uptake and accumulation, and promoting M2 macrophage expression (18).

The mammalian target of rapamycin (mTOR) is a serine/threonine kinase, composed of two distinct scaffold complexes, mTOR Complex 1 (mTORC1) and mTOR Complex 2 (mTORC2), positioned downstream in the PI 3 K/AKT/mTOR signaling pathway. It serves as a critical node in regulating energy supply, biosynthesis, glycolysis, and lipid metabolism. mTORC1 primarily enhances protein synthesis, lipogenesis, energy metabolism, autophagy inhibition, and lysosome formation. Conversely, mTORC2 plays a crucial role in cellular cytoskeletal organization, cell survival, and metabolism (25). TORC1 regulates the polarization of M1 macrophages and the reprogramming of metabolism. Within M1 macrophages, the mTORC1/HIF-1α axis is essential for the transcription of pro-inflammatory cytokines and metabolic genes associated with glycolysis. Studies indicate that mTORC1 influences glycolysis, the pentose phosphate pathway, and lipid metabolism by activating the transcription of genes related to hypoxia-inducible factors and sterol regulatory element-binding proteins. Additionally, FOXK1 independently modulates mTORC1 signaling and CCL2 expression, distinct from NF-κB pathways, thereby promoting tumor progression through the secretion of CCL2 (25). Macrophages activated towards the M2 phenotype primarily utilize fatty acid oxidation and oxidative phosphorylation (OXPHOS) as their chief metabolic pathways, while simultaneously enhancing glucose utilization. Within this framework, mTORC2 operates in parallel with the IL-4Rα/STAT6 pathway, facilitating the augmentation of glycolysis during M2 activation by inducing the transcription factor IRF4.Research indicates that pathogens regulate amino acid metabolism via mTOR, which serves as a critical sensor for amino acids, with its activation contingent upon the abundant availability of certain amino acids. Multiple studies have demonstrated that in innate immune cells such as macrophages, the utilization and metabolism of amino acids are intimately associated with cellular activation and polarization, proceeding in a manner dependent on mTOR signaling.” Furthermore, pathogens such as Leishmania, Plasmodium, Candida albicans, Salmonella enterica, and Helicobacter pylori (RocF) express their own arginases to promote the production of polyamines rather than nitric oxide (NO).This indicates a common strategy among pathogens to evade the deleterious effects of NO. Additionally, the synthesis of polyamines itself has been shown to enhance the anti-inflammatory alternative activation (AA) program in macrophages, as it facilitates mitochondrial respiration, which underpins this type of macrophage polarization (54). Although promoting this AA phenotype might impede the direct clearance of pathogens, AA macrophages play a crucial role in tissue repair, thereby providing significant protection against tissue damage caused by these pathogens in the host.

In summary, as our understanding of the macrophage metabolic-immunoregulatory networks deepens, modulating these signaling pathways and optimizing the metabolic states of macrophages hold promise as effective strategies for controlling and treating chronic infectious diseases.

## 3 Pathogen regulation of macrophage metabolic reprogramming to reshape the immune microenvironment in chronic infections

In chronic infections, pathogens regulate macrophage metabolic reprogramming not only to control their functional expression but also to modulate interactions between macrophages and other immune cells through nutrient competition, metabolite exchange, and signal transduction. This reshapes the immunosuppressive microenvironment, facilitating immune evasion. Additionally, macrophages can increase the production of inflammatory factors through continuous metabolic changes, a process known as trained immunity.

### 3.1 Macrophage metabolic reprogramming suppresses natural killer cell activity in chronic infections

Natural killer (NK) cells, which are members of the innate lymphoid cells (ILCs) family, represent the initial line of defense in the immune system. In numerous chronic disease models, an imbalanced expression of NK cell receptors has been observed to result in a reduction in cytotoxic activity and cytokine production, ultimately leading to an immunoinactive state. This process appears to involve macrophage metabolic reprogramming. For example, during the initial stages of infection with Mycobacterium tuberculosis (MTB), macrophages rely on glycolysis to meet their energy demands. However, the excessive glucose uptake during this process results in the production of a large amount of lactate. This not only impairs the utilization of glucose by NK cells but also creates a highly acidic microenvironment due to lactate accumulation, which inhibits NK cell activity and function (55). Furthermore, as the disease progresses, in the later stages of MTB infection, macrophages produce high levels of PGE2 by upregulating the expression of cyclooxygenase-2 (COX2) and prostaglandin E synthase 1 (PGES1) (56). PGE2 exerts a suppressive effect on IFN-γ production and NK cell cytolytic activity (57).

### 3.2 Macrophage metabolic reprogramming delays neutrophil apoptosis in chronic infections

In chronic infections, pathogens regulate macrophage metabolism to create an immune microenvironment that deprives neutrophils of necessary nutrients, thereby inhibiting their bactericidal capabilities. Glutamine and its direct metabolite, glutamate, serve as sources of carbon and nitrogen for the synthesis of biosynthetic precursors involved in various metabolic pathways that activate macrophages. Consequently, glutamine is central to the pro-inflammatory and antimicrobial responses of M1-like polarized macrophages against Mycobacterium tuberculosis. Therefore, in the face of chronic infections, macrophages accelerate the consumption of glutamine within the microenvironment. For instance, MTB has been demonstrated to stimulate neutrophil apoptosis, which facilitates immune evasion (58). Glutamine has been evidenced to defer neutrophil apoptosis; however, glutamine is necessary for macrophages in both the initial and final stages of infection. Furthermore, the addition of glutamine to neutrophils has been observed to enhance their phagocytic capacity and ROS production (59).

### 3.3 Macrophage metabolic reprogramming suppresses T lymphocyte anti-inflammatory responses in chronic infections

Macrophage metabolic reprogramming affects the external microenvironment, thereby influencing T cell immune responses. Following MTB infection, macrophages degrade tryptophan and secrete transforming growth factor β (TGF-β), leading to granuloma formation and an immunosuppressive microenvironment. This prevents macrophages from clearing MTB infection and T cells from entering granulomas to eliminate MTB (60). In granulomas, the activity of indoleamine 2,3-dioxygenase (IDO) in macrophages increases, resulting in the degradation of tryptophan into the immunosuppressive metabolite kynurenine (Kyn) (61). Kynurenine can bind to the aryl hydrocarbon receptor (AHR) in CD4+ T cells, while TGF-β binds to its receptor (TGF-βR). This interaction inhibits glycolysis and interferon γ (IFN-γ) secretion in CD4+ T cells, ultimately inducing T cell apoptosis and reducing the number of pro-inflammatory T lymphocytes (62). Additionally, certain viruses, such as Sendai virus, inhibit the production of nitric oxide (NO) as a mechanism to evade host responses. While initially beneficial, prolonged NO production over time leads to host tissue damage and suppression of the Th1 response (63).

## 4 The impact of macrophage metabolic reprogramming on trained immunity in chronic infections

Our immune system is constantly influenced by microbial encounters and pathogen-associated molecular patterns (PAMPs) from the environment. These interactions can lead to long-term functional changes in innate immune cells, enhancing their responsiveness to secondary challenges, a phenomenon known as trained immunity.

In chronic infections, the metabolic shifts induced by the initial activation of macrophages by pathogens impact the induction of trained immunity in these cells. Studies have shown that glycolysis, glutaminolysis, and cholesterol synthesis pathways are essential for β-glucan-induced trained immunity in monocytes (64). The replenishment of the TCA cycle with glutamine and the accumulation of fumarate integrated immune and metabolic circuits, inducing epigenetic reprogramming in monocytes by inhibiting KDM5 histone demethylases. Additionally, fumarate itself induces an epigenetic program similar to β-glucan-induced trained immunity. Consistent with this, inhibiting glutaminolysis and cholesterol synthesis in mice reduces the induction of trained immunity by β-glucan (65).

Furthermore, a recent study demonstrated that impaired glycolysis in macrophages limits their responsiveness during type 2 inflammation, indicating the functional significance of this metabolic constraint. However, one study not only identified the critical role of glutaminolysis and FAO in establishing LPS-induced innate immune memory in macrophages but also provided evidence that glycolytic activation is dispensable for this process (66). This suggests that while these studies emphasize the crucial role of metabolic regulation in *in vitro* trained immunity, our understanding of how macrophage metabolic reprogramming affects trained immunity is still incomplete.

In recent years, some epidemiological and experimental studies have determined that trained immunity has beneficial effects due to heterologous protection against unrelated pathogens (64). However, innate memory responses may also become maladaptive under chronic infection conditions, such as in atherosclerosis, neurodegeneration, and autoimmunity (67). Research has found that adoptive transfer of trained macrophages leads to increased lung inflammation and impaired bacterial clearance after Streptococcus pneumoniae infection, compared to recipients of control macrophages (66). Based on previous descriptions, LPS-mediated reprogramming upon exposure to LPS may affect other lung-resident cell populations, thereby improving pneumonia outcomes.

Therefore, future research on chronic infections should not only focus on the mechanisms of macrophage metabolic reprogramming affecting macrophage polarization but also on how macrophage metabolic reprogramming interacts with other immune cells to reshape the immune microenvironment.

## 5 Discussion and prospect

In this article, we focus on the functional alternation of macrophages in different organs in response to pathogen invasion with changes in the metabolic microenvironment, and the proportion of macrophages in different organs changes with disease progression in order to maintain the dimensionality of the internal environment. This dynamic shift highlights the critical role of macrophages in disease pathogenesis, making them attractive targets for therapeutic interventions. Notably, in many instances, the requirement of metabolic states to regulate functional features of macrophages does not appear to be generalizable, but instead dependent on their particular activity in their homing organ (29). However, most of our understanding of macrophage metabolism in different organs in the context of chronic infection comes from studies on mice, and it has been reported that there are species-specific differences in metabolic adaptation between humans and mice (31, 68). The study of human tissue-resident macrophages by combining metabolic profiling (e.g., transcriptome, proteome, and metabolome) with specific mouse models for functional studies will help fill these knowledge gaps. Understanding its specific metabolic biology has powerful therapeutic potential to control its activity at a specific location. In addition, How does metabolic reprogramming of macrophages differ across latency types, and what are the key metabolic regulators involved at each latency stage? This new perspective may lead to a deeper understanding of the mechanisms related to macrophage polarization and metabolic microenvironment in chronic infection (69).
